# Myt1 overexpression mediates resistance to cell cycle and DNA damage checkpoint kinase inhibitors

**DOI:** 10.3389/fcell.2023.1270542

**Published:** 2023-11-02

**Authors:** Sargun Sokhi, Cody W. Lewis, Amirali B. Bukhari, Joanne Hadfield, Edric J. Xiao, Jeremy Fung, Yea Jin Yoon, Wen-Hsin Hsu, Armin M. Gamper, Gordon K. Chan

**Affiliations:** ^1^ Department of Oncology, University of Alberta, Edmonton, AB, Canada; ^2^ Experimental Oncology, Cross Cancer Institute, Edmonton, AB, Canada; ^3^ Cancer Research Institute of Northern Alberta, University of Alberta, Edmonton, AB, Canada

**Keywords:** cell cycle, cell cycle checkpoint kinase inhibitors, DNA damage checkpoint kinase inhibitors, Myt1 overexpression, resistance

## Abstract

Cell cycle checkpoint kinases serve as important therapeutic targets for various cancers. When they are inhibited by small molecules, checkpoint abrogation can induce cell death or further sensitize cancer cells to other genotoxic therapies. Particularly aberrant Cdk1 activation at the G2/M checkpoint by kinase inhibitors causing unscheduled mitotic entry and mitotic arrest was found to lead to DNA damage and cell death selectively in cancer cells. Promising drugs inhibiting kinases like Wee1 (Adavosertib), Wee1+Myt1 (PD166285), ATR (AZD6738) and Chk1 (UCN-01) have been developed, but clinical data has shown variable efficacy for them with poorly understood mechanisms of resistance. Our lab recently identified Myt1 as a predictive biomarker of acquired resistance to the Wee1 kinase inhibitor, Adavosertib. Here, we investigate the role of Myt1 overexpression in promoting resistance to inhibitors (PD166285, UCN-01 and AZD6738) of other kinases regulating cell cycle progression. We demonstrate that Myt1 confers resistance by compensating Cdk1 inhibition in the presence of these different kinase inhibitors. Myt1 overexpression leads to reduced premature mitotic entry and decreased length of mitosis eventually leading to increased survival rates in Adavosertib treated cells. Elevated Myt1 levels also conferred resistance to inhibitors of ATR or Chk1 inhibitor. Our data supports that Myt1 overexpression is a common mechanism by which cancer cells can acquire resistance to a variety of drugs entering the clinic that aim to induce mitotic catastrophe by abrogating the G2/M checkpoint.

## 1 Introduction

The cellular genome is constantly under the threat of exogenous (genotoxic compounds, ultraviolet and ionizing radiation) and/or endogenous (replication errors, reactive oxygen species) DNA damaging agents (Vasan et al., 2019; Bukhari et al., 2022; André A Costa et al., 2023). To counteract DNA damage or replication stress, normal cells have G1/S, intra-S, and G2/M DNA damage checkpoints and DNA repair pathways together defined as the DNA damage response (DDR) (Pilié et al., 2019; Carusillo and Mussolino, 2020). The inherent genomic instability of tumor cells due to dysregulation of the DDR pathway can result in defects in checkpoint kinases, replication fork restart, or DNA repair (Pilié et al., 2019; Carusillo and Mussolino, 2020; Fedak et al., 2021; Shao et al., 2021). This inherent genomic instability of tumor cells provides a vulnerability that can be exploited. Particularly inhibition of kinases regulating the G2/M checkpoint can force cells to enter mitosis with damaged DNA leading to cancer cell death (Aarts et al., 2012; Do et al., 2013; Schmidt et al., 2017; Lewis et al., 2019; Bukhari et al., 2022). The G2/M (DNA damage) checkpoint kinases are potential therapeutic targets because cancer cells have a defective G1/S checkpoint making them more reliant on the G2/M checkpoint. Selective inhibitors against Wee1 (Adavosertib), ATR (AZD6738), Chk1 (UCN-01) that guard these checkpoints have entered phase I/II clinical trials often in conjunction with radiation or other genotoxic agents^1^.

The G2/M checkpoint is regulated by Wee1 and Myt1, two partially redundant effector kinases that add inhibitory phosphates to the mitosis promoting complex, Cdk1/cyclin B1 (Fattaey and Booher, 1997; Chow et al., 2011; Chow and Poon, 2012; Do et al., 2013; Schmidt et al., 2017; Lewis et al., 2019; Bukhari et al., 2022). While Wee1 is a nuclear kinase that phosphorylates Cdk1 on tyrosine 15 (Y15) during S and G2/M checkpoint activation, Myt1 is a dual kinase that can phosphorylate Cdk1 on Y15 and threonine 14 (T14). Both phosphorylations are thought to inhibit Cdk1 activity (Mir et al., 2010; Do et al., 2013; Lewis et al., 2017; Lewis et al., 2019; Schmidt et al., 2017; Bukhari et al., 2022). When a cell is ready to enter mitosis, the balance of activities of the phosphatase Cdc25C versus the kinases Wee1/Myt1 is shifted leading to a removal of inhibitory phosphorylations on Cdk1. Intriguingly, Wee1 activity is later again required to normally exit mitosis as it (re)phosphorylates Cdk1 at the end of mitotic phase (Lewis et al., 2017). Due to defects in the G1/S checkpoint and other DDR components, cancer cells are more reliant on the G2/M checkpoint as demonstrated by Wee1 overexpression in various tumors (e.g., glioblastoma and breast cancer) making it an attractive drug target for therapy (De Witt Hamer et al., 2011; Do et al., 2013; Bukhari et al., 2022). Myt1 has also been found to be a highly expressed gene in various malignancies and more recently it has been associated with poor prognosis in TNBC (Shao et al., 2021; Li et al., 2023). Furthermore, Myt1 has emerged as a candidate major player in tumor progression and drug resistance. Overexpression of Wee1 and/or Myt1 helps cells to enforce the DNA damage checkpoint (G2/M) giving cells more time to repair the damage caused by genotoxic therapies. DNA damage checkpoint kinases such as ATR and Chk1 function in a parallel pathway to Wee1 and Myt1 (Maréchal and Zou, 2013; Awasthi et al., 2015). Ataxia telangiectasia and Rad3 related (ATR) is an apical kinase in the DDR pathway that initiates the cellular response to single stranded DNA (ssDNA) arising from replication stress or resected double strand breaks (Gamper et al., 2013; Maréchal and Zou, 2013; Awasthi et al., 2015; Carusillo and Mussolino, 2020; Fedak et al., 2021). Of the many ATR substrates, Chk1 phosphorylation on serine 345 (S345) is particularly important upon DNA damage as it translates into an intra-S or G2/M cell cycle arrest, giving cells time to repair their damaged DNA. Phosphorylated Chk1 inhibits and targets the phosphatase Cdc25 for degradation which in turn increases the phosphorylation status and reduces the activity of Cdk1 (Bukhari et al., 2022). The ATR-Chk1 pathway downregulates Cdk1 activity by inhibiting the Cdc25 phosphatases family or through activation of p53/p21 in response to replication stress (Qiu et al., 2018). Considering their role as tumor suppressors as well as DNA damage response mediators, ATR and Chk1 have also been identified as cancer therapeutic targets (Awasthi et al., 2015; Qiu et al., 2018; Pilié et al., 2019; Carusillo and Mussolino, 2020).

Inhibition of Wee1 with Adavosertib causes premature entry into mitosis, centromere fragmentation and delayed mitotic exit (Lewis et al., 2017). On the other hand, the mechanism of action of Chk1 and ATR inhibitors is at least partially attributed to the upregulation of the activity of phosphatases in the Cdc25 family (Qiu et al., 2018). Both Chk1 and ATR inhibitors induce ectopic Cdk1 activity and cell death (Moiseeva et al., 2019). Nevertheless, clinical studies have shown variable results for these checkpoint kinase inhibitors and the mechanisms of resistance have not been fully explored (Kummar et al., 2010; Fracasso et al., 2011; Gojo et al., 2013; Van Linden et al., 2013; Do et al., 2015; Pilié et al., 2019; Shah et al., 2021). Our lab recently published that Myt1 is a predictive biomarker of intrinsic and acquired resistance to the Wee1 inhibitor Adavosertib (Lewis et al., 2019). Lung cancer cells that acquire Adavosertib resistance have also been reported to have upregulated ATR and Chk1 activity, which suggests that these kinases also play an important role in determining cancer cell sensitivity to Adavosertib (Sen et al., 2017). Based on their effect on Cdc25C and Cdk1, ATR and Chk1 likely counteract the effects of Adavosertib by reducing ectopic Cdk1 activity. Furthermore, Myt1 knockdown accelerates the rate at which cells enter mitosis when combined with Wee1 or Chk1 chemical inhibitors following irradiation (Chow and Poon, 2012). Having found Myt1 to be an important player promoting resistance to Wee1 inhibition and the interplay between these different checkpoint kinases, we speculated that Myt1 could also be an important candidate conferring resistance to the other inhibitors of the kinases in the parallel DDR pathway (Fattaey and Booher, 1997; Wells et al., 1999; Schmidt et al., 2017; Lewis et al., 2019).

Here, we showed that Myt1 overexpression is also a key mediator of resistance to small molecule inhibitors of ATR and Chk1. Using a tetracycline inducible Myt1 overexpressing cell line, we delineate the direct effects of Myt1 overexpression towards the efficacy of the inhibitors. We found that Myt1 overexpression leads to a rescue in the induction of persistent DNA damage and premature entry in mitosis following Adavosertib treatment. It also mitigated the effect of targeting the ATR-Chk1 pathway. In either case it decreased Cdk1 activity. Considering this role of Myt1, we tested a Wee1/Myt1 dual inhibitor, PD166285 (Wang et al., 2001), to investigate if it can overcome the resistance mediated by Myt1 overexpression. PD166285 was previously shown to cause G2 checkpoint abrogation and premature mitotic entry in B16 mouse melanoma cells (Hashimoto et al., 2006). Here, we show that PD166285 treatment leads to a decrease in both pT14-Cdk1 and pY15-Cdk1 levels while partially rescuing the effects of Myt1 overexpression.

## 2 Materials and methods

### 2.1 Cell culture

HeLa, MDA-MB-231, HeLa Flp-In^™^ T-Rex^™^-APEX2-GFP-Myt1 mCherry-H2B, and HeLa Flp-In™ T-Rex™-APEX2-GFP-Myt1 mRuby-H2B cells, were grown as a monolayer in high-glucose DMEM (Dulbecco’s Modified Eagle’s Medium) supplemented with 2 mM L-glutamine and 5% (v/v) FBS (Fetal Bovine Serum) in a humidified incubator at 37°C with 5% CO_2_.

### 2.2 Cell synchronization

For cells synchronized in G1–S phase by double thymidine block, 2 mM thymidine was added to cells for 16 h with an 8-h release between treatments with thymidine, followed by release for 4 h into media containing vehicle or kinase inhibitors (Hadfield et al., 2022). For synchronization by single thymidine block, 2 mM thymidine was added to cells for 16 h followed by release into media with drugs or vehicle.

### 2.3 Small molecule inhibitors

Small molecule inhibitors were stored as 10 mM solutions in DMSO at −20°C. Cells were treated with 2 µM tetracycline (FisherBiotech; 64-75-5), 2 mM thymidine (Sigma; T1895), 500 nM Adavosertib (Chemie Tek; 955365-80-7), 500 nM PD166285 (Selleckchem; S8148), 1 µM UCN-01 (Sigma-Aldrich; 112953-11-4), and 1 µM AZD6738 (provided by AstraZeneca).

### 2.4 Crystal violet assay

HeLa Flp-In^™^ T-Rex^™^-APEX2-GFP-Myt1 mCherry-H2B cells were seeded at a density of 4,000/well in a 96-well plate. HeLa cells were either not treated or treated with 2 µM tetracycline for 24 h and then treated with increasing concentrations (16–2,048 nmol/L, 1:2 serial dilution) of checkpoint kinase inhibitors for an additional 48 h. DMSO was used as a vehicle control. After the drug treatment, media was removed, and the cells were stained with 0.5% crystal violet for 20 min. The dye solution was then removed, and the wells were rinsed 3 times with water and dried. The dye was redissolved in 100% methanol and the absorbance at 570 nm was measured using a FLUOstar OPTIMA microplate reader (BMG Labtech). Graphs show the average percent cell survival normalized to DMSO represented as 100% cell survival. Error bars represent SEM.

### 2.5 Clonogenic assay

For Myt1 overexpression, HeLa Flp-In^™^ T-Rex^™^-APEX2-GFP-Myt1 mCherry-H2B cells were treated with 2 µM tetracycline for 24 h. Prior to cell seeding, GFP expression was confirmed under a fluorescent microscope. 500 cells were seeded in 6 cm plate 4 h prior to treatment with increasing concentrations of AZD6738, Adavosertib, UCN-01, and PD-166285 (100–2,000 nM). To ensure sustained Myt1 expression, cells were maintained in 2 µM tetracycline for the duration of the experiment. Cells not treated with tetracycline but the drugs served as the baseline control. Drugs were washed off 24 h after treatment and cells were replenished with fresh DMEM medium and allowed to grow and form colonies over 14 days. Colonies were then fixed and stained with 0.5% crystal violet (in 70% ethanol) and counted. A colony was defined as a cluster with 50 or more cells.

### 2.6 High content imaging

A high content imaging system (MetaXpress Micro XLS, software version 6, Molecular Devices) was used to determine the mitotic duration of HeLa cells expressing GFP-Myt1 and mCherry-H2B by time-lapse microscopy. Cells were kept in a humidified environment at 37°C with 5% CO_2_ throughout the experiment. Cells were seeded onto 96-well plate and were synchronized in G1/S phase using a double thymidine block and then released into media containing DMSO or Adavosertib with or without tetracycline. Or the HeLa cells with or without tetracycline were synchronized in G1/S phase using a double thymidine block and released into fresh media for 8 h. Post 8 h they were then treated with DMSO or Adavosertib. The cells were imaged for 23/26 h at 10 min intervals using a 20X objective lens after the treatment. Single images were captured in each well with a 20X (NA 0.75) objective with the equipped siCMOS camera using bandpass filters of 536/40 and 624/40 nm. HeLa cells expressing GFPMyt1 and mRuby-H2B were treated with 250 nM Adavosertib in the presence or absence of 2 µM tetracycline and then analyzed by spinning disc time-lapse microscopy as an additional experiment to validate whether GFP-Myt1 can prevent mitotic arrest in the presence of Adavosertib. Timlapse microscopy was performed with spinning disk confocal microscopy as described in Lewis et al. (2019). For all the above experiments, mitotic duration time was analyzed manually from prophase to anaphase/slippage/cell death. The indicator for prophase was the first sign of chromosome condensation and cell death was verified upon observation of nuclear shrinkage, DNA fragmentation, formation of blebs and apoptotic bodies. Statistical analysis was done using GraphPad Prism version 8.0.1. One-way ANOVA was used to determine statistical significance.

### 2.7 Drug treatment for immunoblotting and kinase assay

Cells were seeded in 100 mm diameter dishes, allowed to grow until ∼80% confluency in a humified chamber at 37°C with 5% CO_2_ and then treated with DMSO, Adavosertib (250 and 500 nM), PD166825 (250 and 500 nM), AZD6738 (500 nM), and UCN-01 (1,000 nM). After 4-h treatment with these drugs, cells were then subjected to further processing for immunoblotting or kinase assay.

### 2.8 Kinase assay

Cdk1 kinase assays were completed as outlined in Lewis et al. (2013); Lewis et al. (2017); Lewis et al. (2019). Briefly, 20 ng of GST-PP1Cα fusion protein (PP1Cα peptide: GRPITPPRN) was added to cell lysate (from 2,000 cells) in 2x Cdk1 phospho-buffer (100 mM β-glycerophosphate, 20 mM MgCl2, 20 mM NaF, 2 mM DTT), 400 µM ATP and then incubated at 37°C for 15 min. Reactions were then terminated with Laemmli sample buffer (BioRad; 161-0747). Levels of pT320-PP1Cα and GST were determined by Western blot. Cdk1 activity was quantified as the ratio of pT320-PP1Cα and GST (total substrate), minus a “no lysate” control (background subtraction). A two-way ANOVA test was used to determine statistical significance.

### 2.9 Immunoblotting

The protein extracts were prepared through the following procedure: cells were washed with PBS and then spun down, lysed with RIPA buffer, and sonicated for 10 cycles 30 s on / 30 s off. Protein levels were standardized with BCA assay and samples were prepared with 2X SDS loading dye with β-mercaptoethanol. Protein extracts were ran on 8% (Wee1, Myt1, tubulin) or 10% (Cdk1, CDK1 pT14, CDK1 -Y15) or 12% (pS345-Chk1, Chk1, GFP-Myt1, pY15-Cdk1, Cdk1, tubulin, pT320-PP1Cα, anti-GST) SDS-PAGE gel for 45–50 min at 200 V. PageRuler Plus Prestained protein ladder (Thermo Fisher Scientific; 26619) was used as a molecular weight marker. Proteins were transferred on to nitrocellulose for 7–15 min at 25 V by Trans-Blot^®^ Turbo Transfer System and blocked in Odyssey blocking buffer (LI-COR Biosciences). Proteins were stained using rabbit anti-phospho-tyrosine 15-Cdk1 (Cell Signaling Technology; 9,111; 1:1,000 dilution), rabbit anti-phospho-threonine 14-Cdk1 (as described in Lewis et al. (2019)); 1:1,000 dilution), mouse anti-Cdc2 p34 (Santa Cruz Biotechnology; sc-54; 1:1,000 dilution), rabbit anti-Wee1 (Cell Signaling Technology; 4,936; 1:1,000 dilution), anti-pT320-PP1Cα antibody (Abcam; ab62334; 1:30,000 dilution), anti-GST antibody (Rockland; 600-401-200; 1:2,000 dilution), rabbit anti-phospho-serine 345-Chk1 (Cell Signaling Technology; 2,348; 1:1,000 dilution), mouse anti-Chk1 (Cell Signaling Technology; 2,360; 1:5,000), anti-tubulin antibody (Sigma; T5168; 1:10,000 dilution), anti-Myt1 antibody (Cell Signaling Technology; 4,282; 1:500 dilution), and anti-GFP IR800-Conjugated antibody (Rockland; 600-132-215; 1:1,000 dilution). Secondary antibodies used were Alexa Fluor 680–conjugated anti-rabbit (Thermo Fisher Scientific; A21109; 1:10,000 dilution), anti-mouse (Thermo Fisher Scientific; A21057; 1:10,000 dilution), IR800 anti-mouse (LI-COR Biosciences; 926-32210; 1:10,000 dilution), and IR800 anti-rabbit (LI-COR Biosciences; 926-32211; 1:10,000). Membranes were imaged using Odyssey Fc (LI-COR Biosciences) scanner and analyzed through Image Studio Lite software version 5.2. For Myt1, anti-rabbit HRP secondary (Cell Signaling Technology 7074S; 1:2,500) was used with ECL Western blotting detection reagent (Cytiva Lifesciences RPN2134; 1:1 dilution, Amersham), imaged using Fuji X-ray film and developed on KODAK M35A X-OMAT Processor. Films were then scanned.

### 2.10 Immunofluorescence

Cells were seeded at a density of 5 × 10^4^ cells/mL on 18-mm^2^ glass coverslips in a 35 mm dish and synchronized by single thymidine block. Following synchronization, cells were released into 500 nM Adavosertib, 500 nM PD166285, 1 μM UCN-01, 1 µM AZD6738, 500 nM Adavosertib +1 µM AZD6738, or equal volume DMSO control for 4 h. Cells were fixed with 3.5% paraformaldehyde in PBS for 7 min, permeabilized in KB buffer (50 mM Tris/HCl, pH 7.4, 150 mM NaCl and 0.1% BSA) with 0.2% Triton X-100 for 5 min at room temperature and rinsed in KB buffer for 5 min at room temperature. Coverslips were stained with anti-phospho-Ser10 Histone H3 (PH3) antibodies (1:1,000 dilution; Abcam; ab5176) and 0.1 μg/mL DAPI. Secondary antibody Alexa Fluor 488-conjugated anti-rabbit (1:1,000 dilution; Thermofisher; A11008) was used. Coverslips were mounted with 1 mg/mL Mowiol 4-88 (EMD Millipore). A Leica Falcon SP8 microscope with a 25 × 0.95 Water HC Fluotar lens was used to collect the images. Diode 405 nm and White laser 2 were used. The white light laser was adjusted for 488 nm and 555 nm. Laser power was kept consistent between image acquisition within each experiment. Images were processed using Adobe Photoshop CS6. The number of PH3 positive cells and total cells was manually counted. The proportion of cells positive for PH3 was calculated and statistical significance was determined by Student’s t-test. A minimum of 500 cells were counted per treatment.

## 3 Results

### 3.1 Myt1 overexpression in HeLa cells does not affect cell division in non treated cells

Previously, we showed that transient overexpression of GFP-Myt1 in HeLa and MDA-MB-231 cells promoted Adavosertib resistance (Lewis et al., 2019); GFP-Myt1 reduced *in vitro* Cdk1 activity and promoted cell survival in Wee1 inhibited cells. However, we were unable to test the effects of GFP-Myt1 overexpression on mitosis directly due to high cytotoxicity induced by transfection. Furthermore, transfection efficiencies varied between experiments, and we were unable to generate a stable cell line. To overcome these technical issues, a tetracycline inducible (Tet-On) Myt1 system was developed using Flp-In^™^ T-Rex^™^ HeLa cells. Using an inducible system ensures the optimal comparison between endogenous and overexpressed Myt1 levels in the same cell populations and avoids potential artifacts due to clonal populations of stable cell lines derived from a heterogeneous cell population as well as phenotypic drifts caused by constitutive overexpression. The cell line established depicted tight regulation of Myt1 overexpression only upon tetracycline addition. To validate our inducible Myt1 system, Flp-In^™^ T-Rex^™^ HeLa cells containing GFP-Myt1 were treated with or without 2 µM tetracycline. Within 24 h of tetracycline treatment, we observed ubiquitous GFP-Myt1 expression by immunoblot and fluorescence microscopy (Supplementary Figures S1A, B); however, in the absence of tetracycline, GFP-Myt1 levels were undetectable. Given the functional role of Myt1 in Cdk1 inhibition, Myt1 overexpression may induce a cell cycle arrest (Mueller et al., 1995; Liu et al., 1999). To confirm that GFP-Myt1 overexpression did not induce a cell-cycle arrest in our system, we treated cells with tetracycline for 48 h and then fixed and stained cells with pS10-histone H3 (PH3), a marker of mitosis, and DAPI (Supplementary Figures S1C, D). Similar proportions of PH3 positive cells were observed in the cell populations with or without tetracycline, confirming that GFP-Myt1 at the observed levels does not induce a cell-cycle arrest in interphase. Furthermore, mitotic cells overexpressing GFP-Myt1 exhibited normal chromosome condensation and segregation (Supplementary Figure S1E).

GFP-Myt1 was enriched in globular structures less than 2 µm in size outside the nucleus during interphase (Supplementary Figure S1E; top two panels). The structures in question were absent in mitosis (Supplementary Figure S1E; bottom three panels). This may suggest that these structures are disassembled during the onset of mitosis and then reassemble following mitosis. Furthermore, similar structures were previously observed by our lab during transient transfection with an alternative GFP-Myt1 plasmid lacking APEX2 (pEGFP-Myt1-GW) in HeLa and MDA-MB-231, confirming that the formation of these structures is not unique to the Flp-In^™^ T-Rex^™^ GFP-APEX2-Myt1 HeLa system. A further analysis revealed that Myt1 localizes to lipid droplets (manuscript in preparation).

### 3.2 Myt1 overexpression prevents premature mitotic entry from S phase in the presence of adavosertib

**FIGURE 1:**
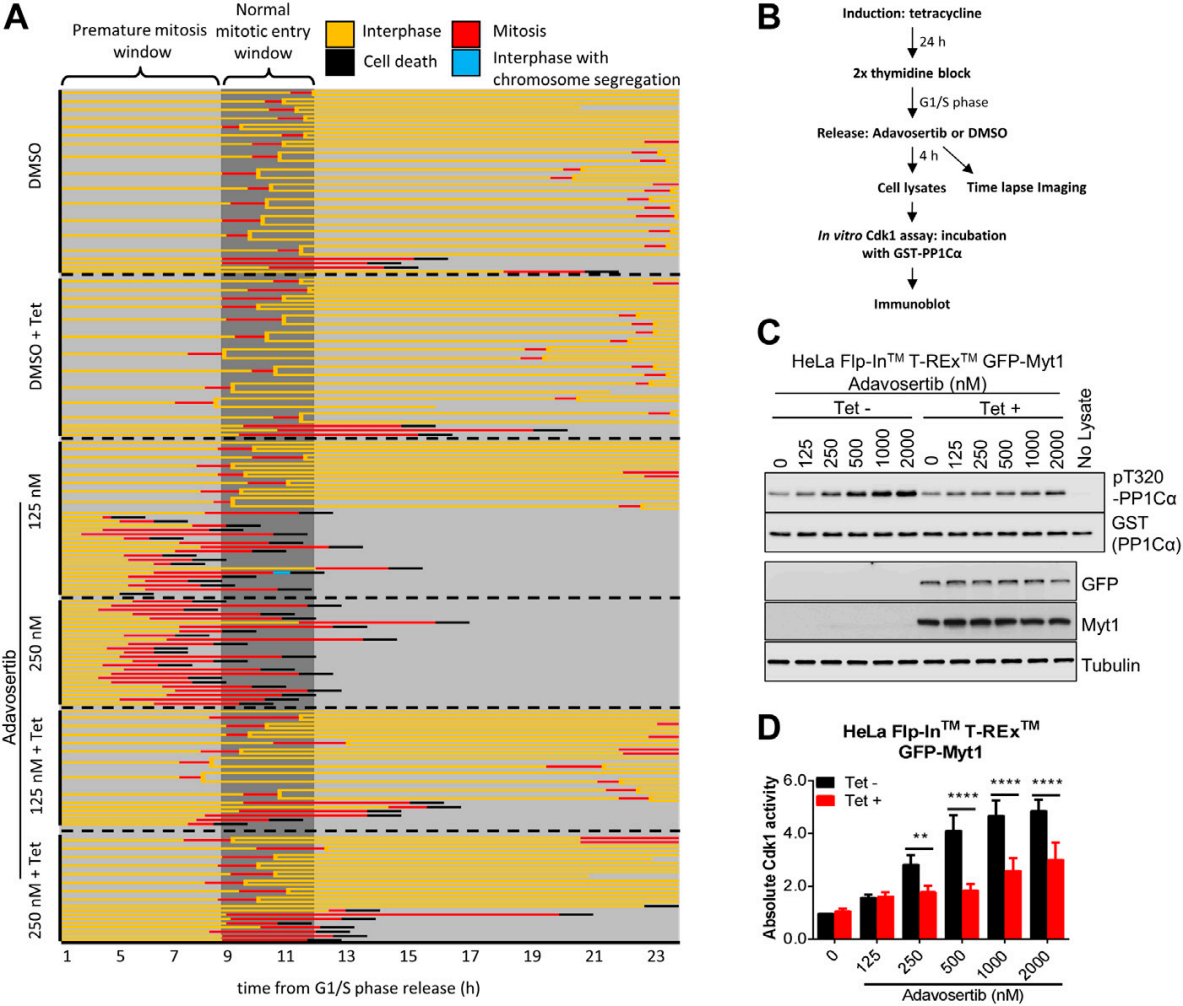
Myt1 overexpression prevents premature mitotic entry from S phase in Adavosertib treated cells. **(A)** Non treated or tetracycline treated HeLa cells were released from G1/S phase into media containing either DMSO or Adavosertib and then observed by time-lapse microscopy (1 h post G1/S release). Each line represents the fate of a single cell and forked lines indicate cell divisions. The dark grey box indicates the time when nontreated cells are expected to enter mitosis (9–11 h). **(B)** The flow chart depicts *in vitro* kinase assay and time lapse imaging protocols. Cdk1 activity in lysates from cells 4 h after G1/S release was assessed *in vitro* by incubation with GST-PP1Cα (a Cdk1 substrate). Total pT320-PP1Cα peptide and GST levels were determined by immunoblot. **(C)** *In vitro* Cdk1 activity (top two panels) was assessed in HeLa cells (treatments are indicated). GFP, Myt1 (band for the fusion protein is shown), and tubulin levels were analyzed by immunoblot (bottom three panels). **(D)** The graph shows the quantitation of average Cdk1 activity (relative to control Tet-/DMSO). Error bars represent SEM. ** and **** denote *p* < 0.01 and *p* < 0.0001 (Two-way ANOVA). Experiments were repeated at least three times.

We previously showed that Adavosertib disrupts the cell cycle by at least two independent mechanisms: forced mitotic entry from S phase and delayed mitotic exit (Lewis et al., 2019). To test if GFP-Myt1 overexpression prevented Adavosertib treated cells from entering mitosis directly from S phase, cells were pretreated with tetracycline to modulate Myt1 levels, synchronized in G1/S by a double thymidine block and released into fresh media containing either DMSO or Adavosertib (Figure 1B). The cell fate, including the point at which cells enter mitosis, was then analyzed by time-lapse microscopy (Figure 1A). Most DMSO-treated cells, with or without tetracycline, entered mitosis 9–11 h post G1/S release (Figure 1A; top two panels). On the other hand, non-induced cells treated with 125–250 nM Adavosertib entered mitosis within 3–5 h, indicating premature mitotic entry (Figure 1A; third and fourth panel). In contrast, GFP-Myt1 overexpressing cells treated with Adavosertib entered mitosis after a similar period to that of DMSO controls, which suggests Myt1 prevents premature mitotic entry in the absence of Wee1 activity (Figure 1A; 5^th^ and 6^th^ panel). To further corroborate that the effect is mediated via Cdk1, the *in vitro* Cdk1 activity was measured of lysates obtained 4 h after releases from G1/S phase (Figures 1C, D). Consistent with the imaging experiments, GFP-Myt1 overexpressing cells exhibited significantly lower *in vitro* Cdk1 activity relative to non-overexpressing cells in the presence of Adavosertib.

### 3.3 Myt1 overexpression promotes mitotic exit in Wee1 inhibited cells and reduces the chance of mitotic catastrophe

**FIGURE 2:**
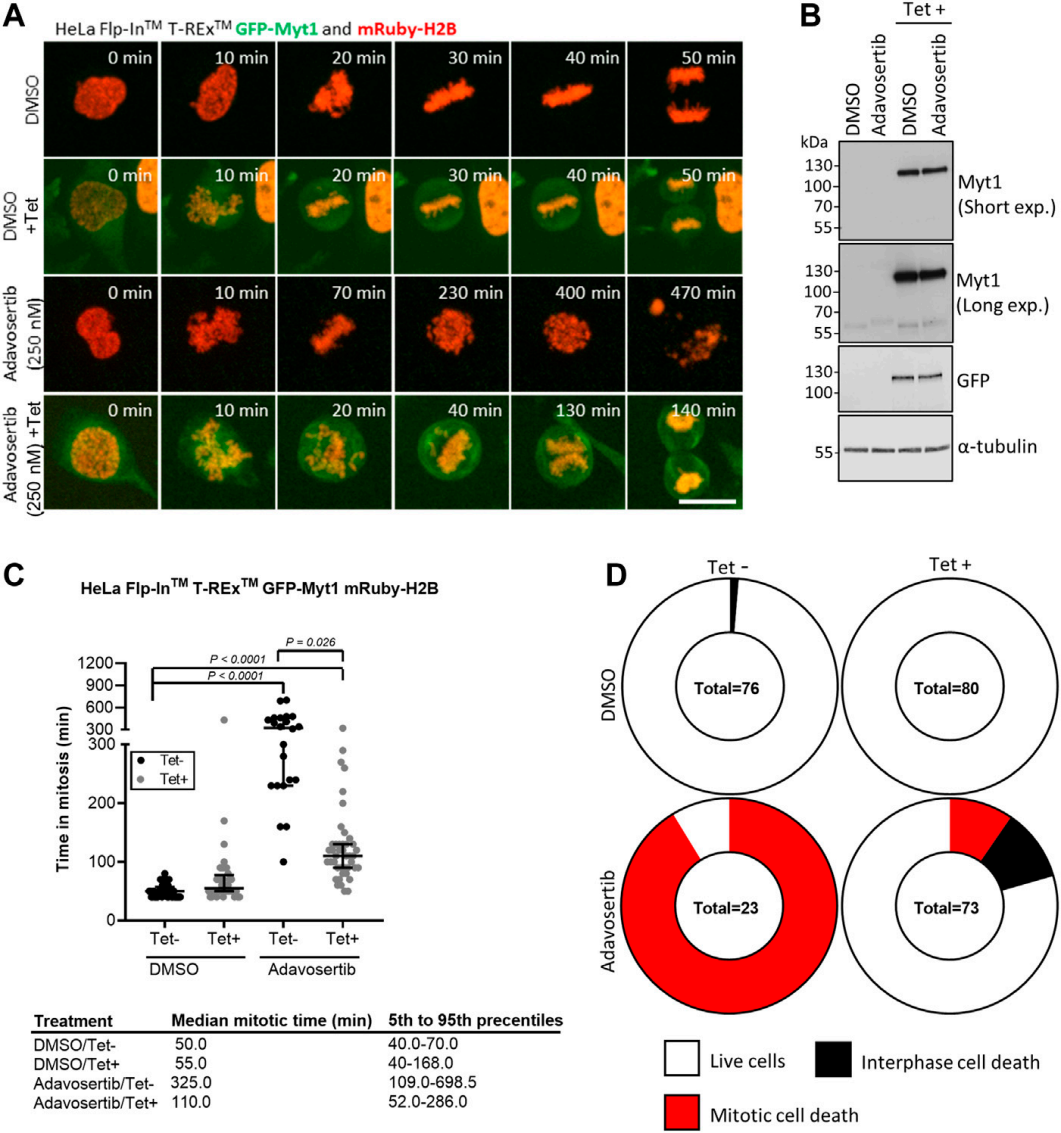
Myt1 overexpression rescues cells from Adavosertib induced mitotic arrest. **(A)** HeLa cells were treated with 250 nM Adavosertib in the presence or absence of 2 µM tetracycline and then analyzed by time-lapse microscopy. Scale bar = 10 µm. **(B)** An immunoblot shows total levels of GFP, Myt1, and tubulin. **(C)** Time in mitosis for indicated treatments is shown. **** denotes *p* < 0.0001 (ANOVA). Median mitotic times are included in the table below. **(D)** Donut charts show the proportion of cell death for each treatment. The number of cells counted is shown within each chart. Experiments were repeated at least three times.

Next, we tested if GFP-Myt1 overexpression could rescue cells from aberrant mitosis induced by Adavosertib. We first established stable Flp-In^™^ T-Rex^™^ GFP-Myt1 cells expressing also mRuby-H2B. Asynchronous cell populations treated with either DMSO or 250 nM Adavosertib (with or without tetracycline) were observed by time-lapse microscopy (Figures 2A–C). Overexpressing and non-overexpressing cells had similar median mitotic times (55 min and 50 min). Furthermore, GFP-Myt1 overexpressing cells did not display any defects in chromosome alignment or segregation (Figure 2A). In contrast, nearly all Adavosertib-treated cells were unable to achieve chromosomal alignment in the absence of tetracycline; instead, cells arrested in prometaphase for several hours and then died without completing mitosis (325 min) (Figures 2A, C, D). However, GFP-Myt1 overexpression permitted chromosomal alignment and mitotic exit (110 min) even in the presence of Adavosertib leading to a substantial reduction in mitotic timing and overall cell death (Figures 2A, C, D).

**FIGURE 3:**
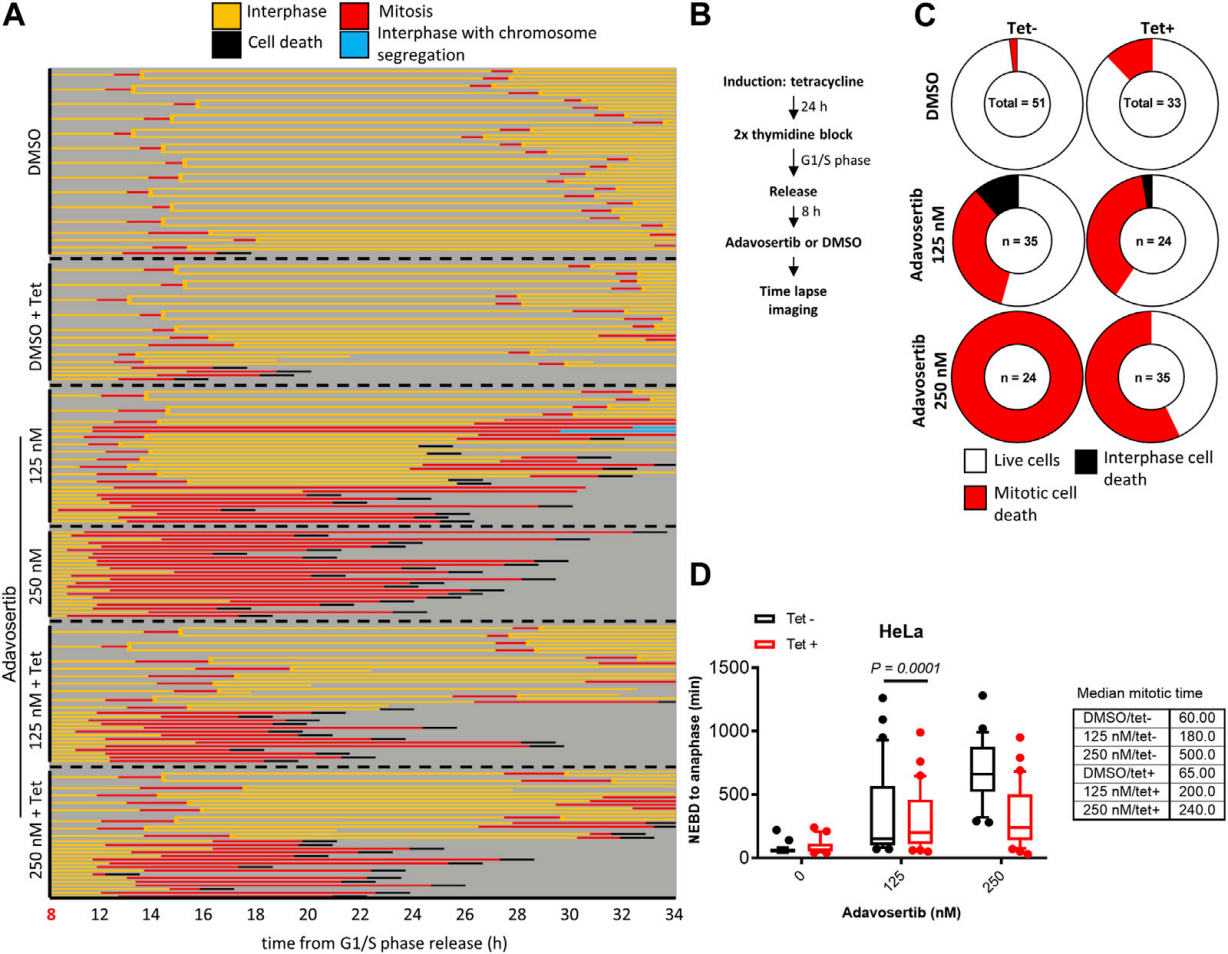
Myt1 overexpression promotes mitotic exit in the presence of Adavosertib. **(A)** Nontreated and tetracycline treated HeLa cells were released from G1/S for 8 h release in fresh medium before adding DMSO or Adavosertib and then analyzed by time-lapse microscopy. Each line represents a single cell and forked lines indicate cell division. **(B)** The flow chart depicts the time lapse imaging protocol. **(C)** Donut charts indicate the proportion of cell death observed for each treatment. The number of cells counted is indicated within each donut plot. **(D)** Graph indicates the duration of mitosis (NEBD to anaphase/mitotic slippage). Median mitotic times are included in the table provided. Experiments were repeated at least three times.

Next, we tested if Myt1 could facilitate mitotic exit in Wee1-inhibited cells that were not prematurely forced into mitosis by Adavosertib. Cells with or without Myt1 overexpression were synchronized in G1/S phase. Only after an 8 h release from the block with fresh media–and thus not prematurely entering mitosis-were the cells treated with Adavosertib and observed by time-lapse microscopy (Figures 3A, B). Most DMSO-treated cells ( ± tetracycline) showed nuclear envelope breakdown (NEBD) and chromosome segregation within 60–65 min, consistent with a normal mitotic duration for HeLa cells (Lewis et al., 2017) (Figure 3A; first two panels). In contrast, non-induced cells that were treated with the increasing concentration of Adavosertib (125–250 nM) were more prone to prometaphase arrest, prolonged mitosis, and cell death in a dose dependent manner (Figures 3A, C, D). Notably, GFP-Myt1 overexpression decreased the number of cells that arrested in prometaphase and died in mitosis due to Wee1 inhibition (effects clearly distinguishable at 250 nM), which suggests that a high Myt1 activity can compensate for the role of Wee1 in promoting mitotic exit (Figure 3A; 5^th^ and 6^th^ panel and Figure 3C).

### 3.4 Inhibitors of kinases regulating checkpoint activation are less cytotoxic towards cancer cells overexpressing Myt1

**FIGURE 4:**
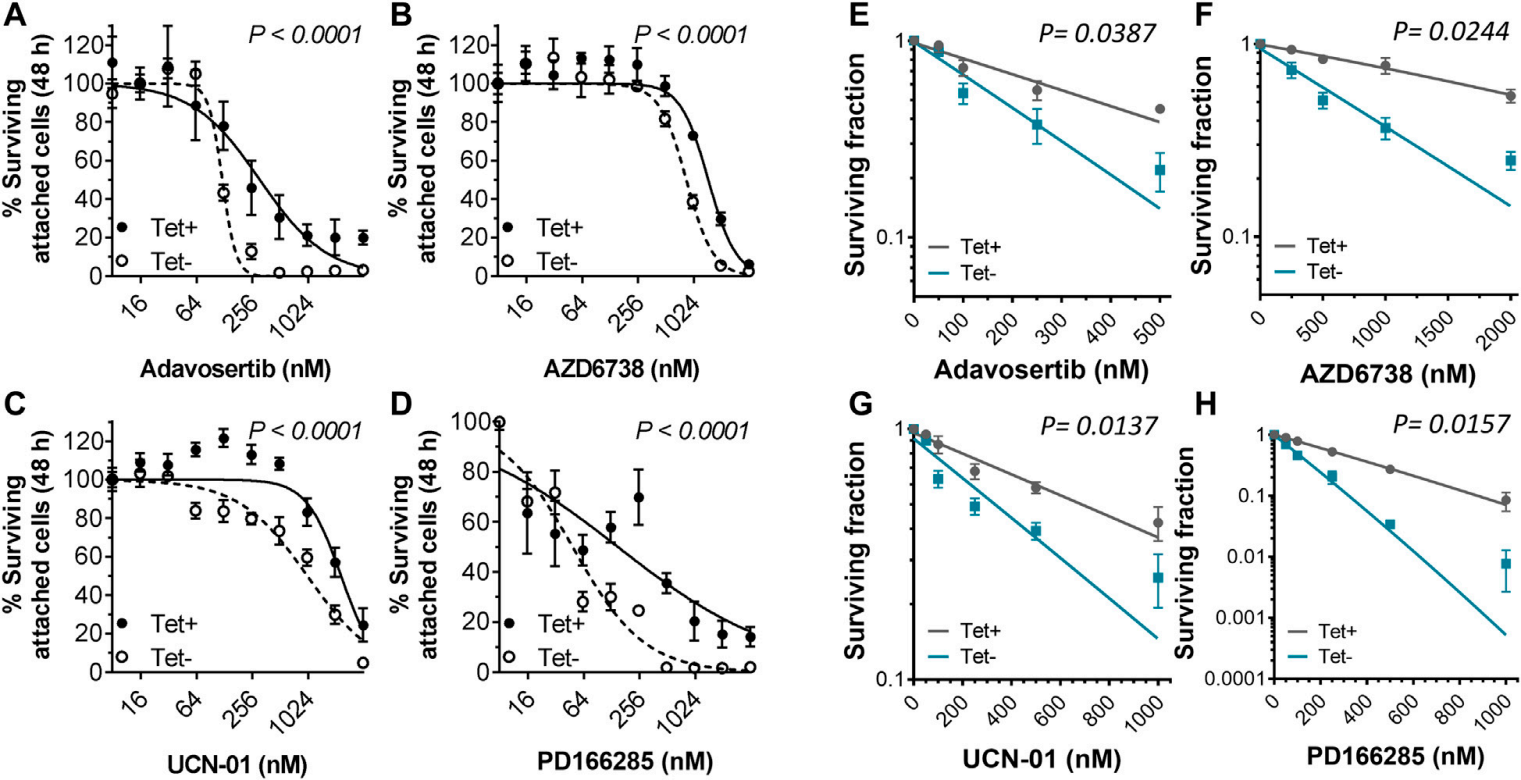
Myt1 overexpression promotes resistance to DNA damage and cell cycle checkpoint kinase inhibitors. HeLa cells were either not treated or treated with 2 µM tetracycline for 24 h and then exposed to **(A)** Adavosertib (500 nM), **(B)** AZD6738 (1000 nM), **(C)** UCN-01 (1000 nM), or **(D)** PD166285 (500 nM) for an additional 48 h. Graphs show the average percent cell survival determined by crystal violet assay. For colony formation assays, HeLa cells with or without tetracycline were treated with **(E)** Adavosertib, **(F)** AZD6738, **(G)** UCN-01, or **(H)** PD166285 for 24 h and then replenished with fresh media to be incubated for 14 days to evaluate their clonogenic potential as shown. Error bars represent SD. Experiments were repeated three times.

To test the sensitivity of tetracycline inducible HeLa cells with or without Myt1 overexpression, cells were either conditioned with or without 2 µM tetracycline for 24 h and then treated with various concentrations of Adavosertib, PD166285, AZD6738 or UCN-01 for 48 h. Cell viability was then evaluated using crystal violet assays. As expected, Myt1 overexpression led to an increase in the IC50 for Adavosertib to 308 nM from 120 nM, compared to HeLa cells with endogenous Myt1 levels (*p* < 0.0001) (Figure 4A). Importantly, tetracycline pretreatment also caused an increase in the survival chance of cells treated with kinase inhibitors targeting ATR, Chk1 or Wee1/Myt1 (*p* < 0.0001) (Figures 4B–D). The respective IC50 values for these inhibitors have been provided in Table 1. Next, we tested whether Myt1 overexpression also promotes the survival specifically of clonogenic cells treated with these kinase inhibitors. The ability of clonogenic cells to (in theory) proliferate indefinitely makes these subpopulation candidate tumor cells capable of metastasizing or leading to tumor recurrence if surviving treatment. Clonogenic survival is thus a better predictor for clinical tumor control than cell proliferation assays. Therefore, tetracycline conditioned or unconditioned cells were seeded and treated with kinase inhibitors for 24 h. After replenishing with fresh media, the cells were then incubated for 14 days before visualizing colonies formed by crystal violet staining (Gamper et al., 2013). The surviving fraction decreased with increasing concentrations of Adavosertib, AZD6738, UCN-01 or PD166285 (Figures 4E–H). However, Myt1 overexpression substantially reduced the cytotoxicity of all four drugs targeting kinases regulating Cdk1 activity as evidenced by the increase in colony formation in plates with cells pretreated with tetracycline compared to vehicle control (Figures 4E–H).

**TABLE 1:** Myt1 overexpression promotes resistance to DNA damage and cell cycle checkpoint kinase inhibitors. The table provides the IC50 values towards different cell cycle or DNA damage checkpoint kinase inhibitors calculated over 48 h either in the presence or absence of tetracycline. The percent cell survival was evaluated using crystal violet assay and the IC50 provided is in nM with a corresponding 95%CI. The experiment was repeated three times.

Treatment	IC50 post 48 h in nM (95%CI)	
	Tet-	Tet+
Adavosertib	120 (114–124)	308 (231–419)
PD166285	48 (40–58)	147 (107–202)
AZ6738	846 (766–931)	1,467 (1,305–1,651)
UCN-01	1,000 (848–1,177)	2,320 (1956–2,776)

### 3.5 Myt1 overexpression inhibits mitotic entry by reducing Cdk1 activity in cells treated with inhibitors of upstream kinases

**FIGURE 5:**
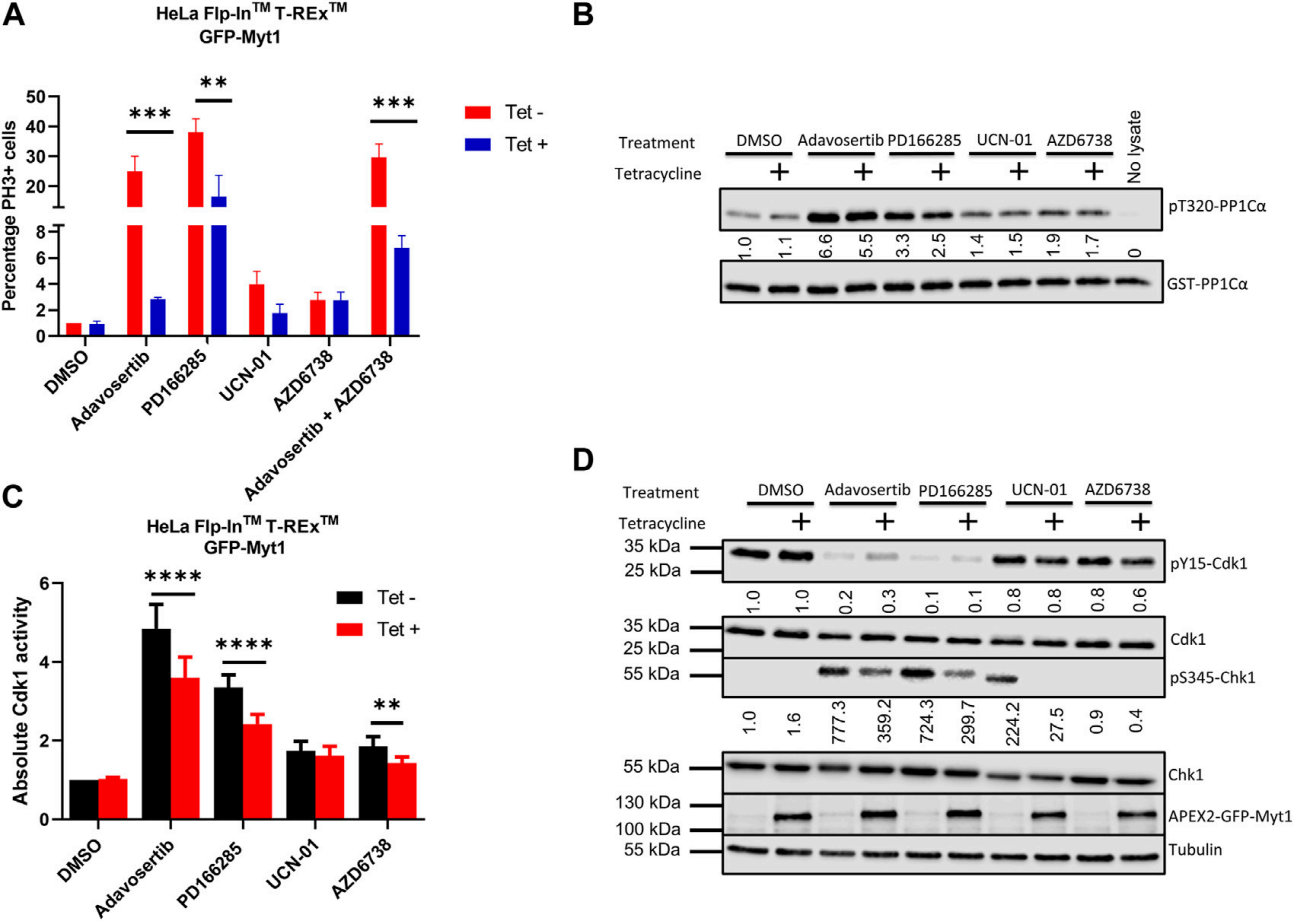
Myt1 overexpression inhibits mitotic entry by reducing Cdk1 activity in cells treated with checkpoint kinase inhibitors **(A)** HeLa cells were seeded onto coverslips in the presence or absence of tetracycline (2 µM) for 24 h. Cells were synchronized in G1-S phase by single thymidine block. Following synchronization cells were released into media containing kinase inhibitors or DMSO for 4 h. Cells were stained for DNA and PH3 and analyzed by immunofluorescence microscopy. The percentage of cells positive for PH3 is shown normalized to DMSO/Tet-. Error bars represent SEM. Experiments were repeated three times. Statistical significance was determined by 2-way ANOVA. ***, *p* < 0.001; **, *p* = 0.001. **(B)** Cell lysates were prepared from tetracycline inducible Myt1 expressing HeLa cells. Cells were treated or not with 2 µM of tetracycline 48 h prior to any drug treatment/control: DMSO, Adavosertib (500 nM), PD166285 (500 nM), UCN-01 (1000 nM), and AZD6738 (1000 nM) for 4 h. Cdk1 activity in lysates from cells was assessed *in vitro* by incubation with GST-PP1Cα (a Cdk1 substrate). Total pT320-PP1Cα peptide and GST levels were determined by immunoblot. **(C)** Bar graphs show the quantitation of the absolute Cdk1 activity in cells treated with various checkpoint kinase inhibitors. Error bars represent SD. Statistical analysis was done using a 2-way ANOVA. ****, *p* < 0.0001**, *p = 0.0029.* **(D)** Lysates were also evaluated for the levels of pY15-Cdk1, Cdk1, pS345-Chk1, Chk1, overexpressed Myt1 and Tubulin. Quantitation shows the average pS345-Chk1 and pY15-Cdk1 levels relative to their total proteins and normalized to DMSO with no tetracycline addition. Kinase assay was repeated 5 times or more. Western blotting and the immunofluorescence experiments were repeated three times.

Cancer cells have dysregulated cell cycle checkpoints (Visconti et al., 2016), which makes them vulnerable to small molecule inhibitors targeting kinases regulating the essential Cdk1. The reliance of many cancer cells on Cdk1 for sustainable proliferation increases the chance of cell death when ectopic Cdk1 activity is promoted. To investigate premature mitotic entry following kinase inhibitor treatment, HeLa cells were either conditioned or unconditioned with 2 µM tetracycline for 24 h, synchronized in G1/S with thymidine, and then treated with the drugs for 4 h after release from the thymidine block as in Figure 1B. The cells were then fixed and stained with DAPI and anti-phospho-histone H3 serine 10 (pS10). 4 h after release from thymidine, HeLa cells are expected to be in S phase, as indeed observed in the DMSO control (Figure 5A and Supplementary Figure S2A). Compared to control the percentage of mitotic (PH3+) cells in the Adavosertib treatment group was much higher (25%) and even more elevated in the group treated with the Wee1/Myt1 dual inhibitor PD166285 (38%). However, Myt1 overexpression caused a significant drop in the percentage of mitotic cells in the Adavosertib and PD166285 treatment group (2% and 16% respectively) (***, *p* = 0.0009 and **, *p* = 0.001). Although we see a slight increase of PH3+ cells post UCN-01 or AZD6738 monotreatment, GFP-Myt1 overexpression is not able to cause a significant drop in PH3+ cells in these treatments. We do see less cells entering premature mitosis following combined treatment of AZD6738 and Adavosertib when comparing cells with overexpressed *versus* endogenous Myt1 levels (30 *versus* 7%; ***, *p* = 0.0007). Yet, the rescue from forced mitotic entry by Myt1 overexpression is reduced in the combination treatment group compared to Adavosertib alone.

To verify that the underlying cause for decreased mitotic entry in Myt1 overexpressing cells is a change in Cdk1 function, we evaluated Cdk1 activity using our *in-vitro* kinase assay. Cell lysates were prepared from tetracycline inducible cells. HeLa cells with or without tetracycline treatment were treated with DMSO, Adavosertib, PD166285, AZD6738 and UCN-01 for 4 h. Then the prepared lysates were incubated with the Cdk1 substrate GST-PP1Cα and the levels of pT320-PP1Cα were quantified by immunoblotting. Wee1 inhibitor Adavosertib, Wee1/Myt1 inhibitor PD166285 and ATR inhibitor AZD6738 significantly increased Cdk1 activity (∼5-fold, 3-fold and 2-fold respectively) compared to DMSO control (Figures 5B, C). However, there is a significant decrease in Cdk1 activity in Adavosertib, PD166285, or AZD6738 treated cells with Myt1 overexpression compared to the respective non-induced cells. There is no significant change in Cdk1 activity in the UCN-01 treatment group between control or Myt1 overexpressing HeLa cells. To determine whether Cdk1 activity correlates to the phosphorylation levels of Cdk1 Y15 and T14, we performed immunoblots with the same set of extracts. As expected, levels of pY15-Cdk1 are decreased by treatment with Adavosertib or even more with PD166285 (Figure 5D). Myt1 overexpression increased phosphorylation of Cdk1 on Y15 in Adavosertib treated cells, but not in PD166285 treated cells. PD166285 inhibits both Myt1 and Wee1, and PD166285 treatment also decreases pT14-Cdk1 levels in addition to pY15-Cdk1 as investigated in HeLa and MDA-MB-231 cells (Supplementary Figure S2B).

To investigate whether Adavosertib or PD166285 treatment activated the ATR signalling pathway, we also tested the phosphorylation of Chk1 at S345, a target site of ATR. Immunoblots of HeLa cell lysate treated with Adavosertib showed strong phosphorylation of Chk1 at S345, indicating induction of genotoxic stress by Wee1 inhibition (Figure 5D). Also, the treatment of HeLa cells with PD166285 activated ATR, maybe even further than Adavosertib. As observed also by others, Chk1 inhibition leads to ATR activation (A shift in the signal in the pS345 Chk1 band compared to the previous lane is likely due to the inhibition of Chk1 autophosphorylation by UCN-01) (Okita et al., 2012). Adavosertib, UCN-01 and PD166285 all can increase genotoxic stress as indicated by ATR activation, but Myt1 overexpression results in decreased pS345-Chk1 levels in each case. It is therefore likely that Myt1 overexpression partially counteracts processes initiated by Wee1 or Chk1 inhibition leading to single stranded DNA and subsequent ATR activation.

## 4 Discussion

**FIGURE 6:**
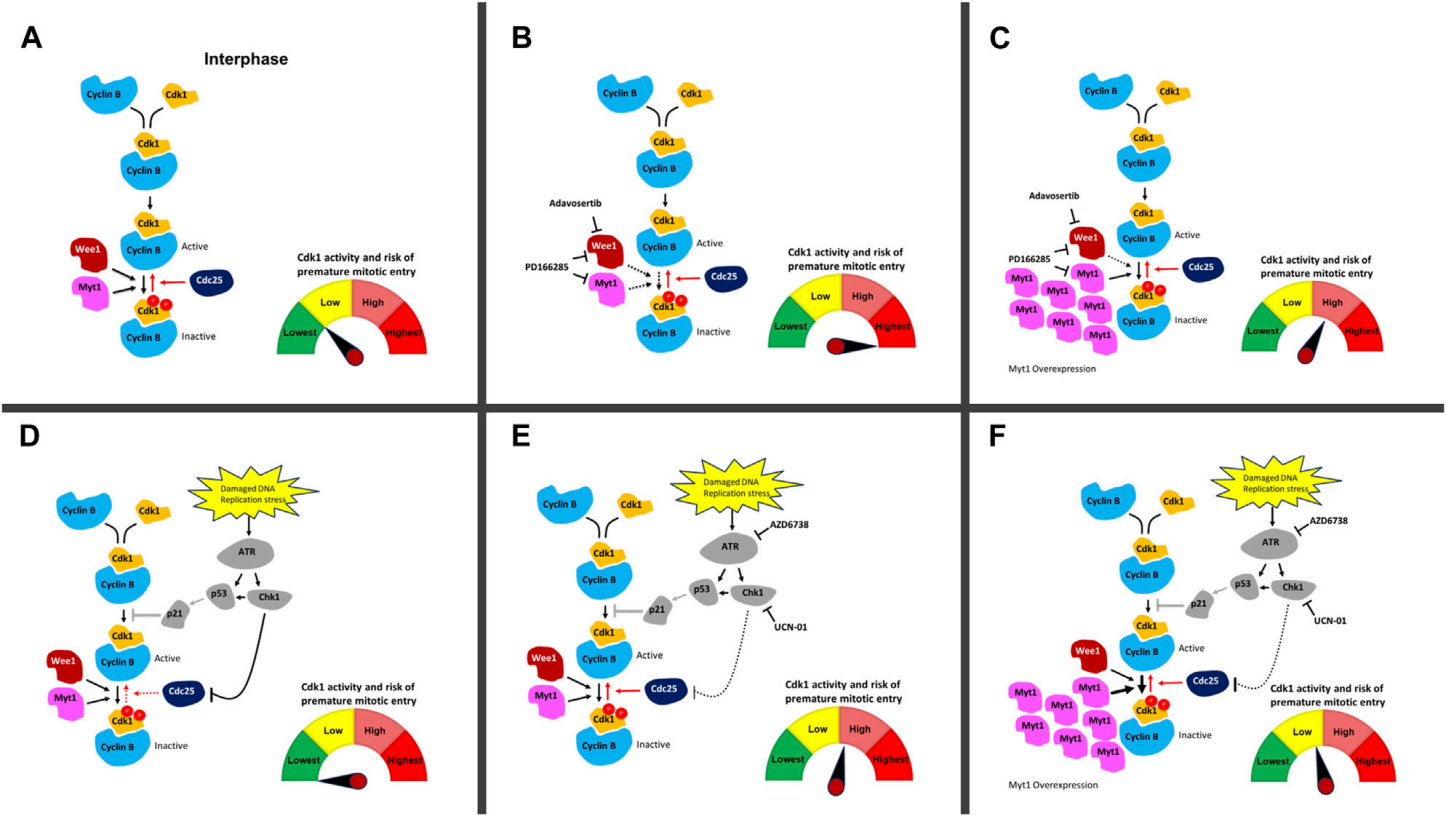
DNA damage and cell cycle checkpoint inhibitors and their effect on ectopic Cdk1 activation. **(A)** In an unperturbed cell, Cdk1 and cyclin B bind in late S-phase. Phosphorylation of Cdk1 on T14 and Y15 (depicted with red circles) by Myt1 and Wee1 during interphase inhibits Cdk1/cyclin B. Cdc25 removes these inhibitory phosphorylations and activates the Cdk1/cyclin B complexes allowing cells to enter mitosis. The high level of Wee1 activity in many cancer cells prevents their premature entry into mitosis and facilitates their survival in the presence of elevated genotoxic stress compared to normal cells. **(B)** Upon addition of Adavosertib (inhibition of Wee1) or PD166285 (inhibition of Wee1 and Myt1), the balance between inactive and active Cdk1/cyclin B1 complexes is shifted leading to more active Cdk1/cyclin B1 complexes. This increases the chance of premature entry into mitosis and cell death. **(C)** Overexpression of Myt1 in the presence of Adavosertib or PD166285 counters the drugs’ effect because phosphorylation of Cdk1 on T14 and Y15 by Myt1 increases inactive Cdk1/cyclin B complexes. This results in checkpoint arrest and rescue from cell death even in the presence of these cell cycle checkpoint kinase inhibitors. **(D)** Damaged DNA or replication stress can induce the activation of the DNA damage checkpoint, which downregulates Cdk1/cyclin B activity via Cdc25. ATR activates the effector kinase Chk1, which phosphorylates and inhibits the Cdc25 phosphatase family. In parallel, ATR can induce the stabilization and transcriptional activity of p53 leading to increased expression of the CDK inhibitor p21; however, this pathway is downregulated in many cancers (due to non-functional p53 status of many cancers) (Olivier et al., 2010) **. (E)** Upon addition of UCN-01 or AZD6738 (inhibition of Chk1 and ATR), Cdc25 inactivation in the presence of DNA damage is repressed and the balance between inactive and active Cdk1/cyclin B1 complexes is shifted towards more active Cdk1/cyclin B1 complexes. The cells are at elevated risk of premature mitotic entry. Restoration of Cdc25 activity in the presence of DNA damage is likely to have a lower effect then inhibiting Wee1 and Myt1 with Adavosertib or PD-166285. **(F)** Upon overexpression of Myt1 in the presence of UCN-01 or AZD6738 which leads to more inhibitory phosphorylation of Cdk1 on both T14 and Y15, the pool is reverted to more inactive Cdk1/cyclin B1 complexes even in the presence of these DNA damage checkpoint kinase inhibitors. Red arrows are Cdk1 activating pathways whereas black arrows are Cdk1 inactivating pathways. Solid line shows an active pathway, a dotted line an inhibited pathway. The p53 pathway (grey) is abrogated in most cancers. The meter schematically indicates the balance in active/inactive Cdk1 complexes in each scenario and the associated risk of premature entry into mitosis.

Wee1 inhibition by Adavosertib disrupts the cell cycle by three independent mechanisms. First, Adavosertib treatment in G1 or early S-phase leads to unscheduled replication origin firing (De Witt Hamer et al., 2011; Aarts et al., 2012). Secondly, and importantly for cancer therapy, the upregulation of Cdk1 activity by Wee1 inhibition can force cancer cells to enter mitosis with under-replicated or unrepaired chromosomes leading to mitotic catastrophe and chromosome fragmentation (Figures 6A, B) (Aarts et al., 2012; Duda et al., 2016; Lewis et al., 2017). Third, Cdk1 re-phosphorylation and cyclin B degradation are key steps required for mitotic exit (Jin et al., 1998; Chow et al., 2011; Visconti et al., 2012; 2015; Lewis et al., 2017). Treatment with Adavosertib thus leads to mitotic arrest due to delayed exit from mitosis (Lewis et al., 2017; Lewis et al., 2019). We previously showed that siRNA knockdown of Myt1 increased the number of cells that prematurely entered mitosis as well as increased the duration of mitotic arrest in HeLa and MDA-MB-231 cells (Lewis et al., 2019). However, the effects of Myt1 overexpression on mitotic timing in Wee1 inhibited cells were not addressed. Here we demonstrate that Myt1 overexpression prevents premature entry into mitosis and also promotes mitotic exit in cells treated with Adavosertib, ultimately rescuing from cell death (Figures 1–3). We propose a mechanism whereby Myt1 at least partially compensates the inhibitory function of Wee1 by phosphorylation on T14-Cdk1 and Y15-Cdk1 leading to reduced ectopic Cdk1 activity and increased resistance to Adavosertib (Figure 6C). Nevertheless, while Myt1 upregulation is an important resisting factor, it is not the only mechanism able to promote resistance to Adavosertib. A previous study has shown that upregulation of receptor tyrosine kinase AXL or downstream targets of AXL and PI3K/mTOR pathways were the strongest markers of resistance to Adavosertib identified in the proteome of a resistant small cell lung carcinoma (SCLC) cell line (Sen et al., 2017). In the same study it was also found that Adavosertib resistance via AXL upregulation was mediated by the ERK/p90RSK signaling cascade, Akt/mTOR signaling and Chk1 activation in SCLC cell lines. Another study in breast cancer cells showed that the loss of PTEN which would enhance replication stress leads to Wee1 inhibition sensitivity (He et al., 2015; Brunner et al., 2020; Bukhari et al., 2022). In addition, the FOXM1 and Cdk1 circuit which is affected by HPV16 oncoproteins, further regulated Wee1 inhibition sensitivity in a head and neck squamous carcinoma model (Diab et al., 2020; Bukhari et al., 2022). This suggests that there are several signaling pathways that can converge on Cdk1 regulation and thus different mechanisms/genetic or epigenetic alterations can lead to Wee1 inhibitor resistance. Further investigation of protein and pathway signatures of the resistant cell lines are warranted to elucidate the interconnectivity underlying the mechanisms leading to resistance.

To further illustrate the effect of Myt1 overexpression on Cdk1 activity as a key mechanism promoting resistance, we used an *in-vitro* kinase assay. We found that Cdk1 activity decreases significantly with Myt1 overexpression in Adavosertib and PD166285 treated cells in comparison to cells with endogenous Myt1 levels treated respectively (Figures 5B, C). Therefore, Myt1 mediates resistance by compensating Cdk1 inhibition in the presence of these inhibitors (Figure 6C, F). To establish a relationship between ectopic Cdk1 activity and premature entry into mitosis, PH3 staining was used to examine premature mitotic entry of synchronized HeLa cells treated with inhibitors of several checkpoint kinases. The treatment with Adavosertib, PD166285, and a combination of Adavosertib and AZD6738 resulted in an increase in percentage of cells undergoing premature mitosis (Figure 5A and Supplementary Figure S2A). The increase in PH3+ cells with Adavosertib treatment is consistent with previous findings that Wee1 inhibition leads to premature mitosis leading to mitotic catastrophe (Lewis et al., 2017; Bukhari et al., 2019; Lewis et al., 2019). The observed premature entry into mitosis with these checkpoint kinase inhibitors is also congruent with the increase in Cdk1 activity observed *in vitro* (Figure 5B, C). With the induction of GFP-Myt1, the percentage of PH3+ cells reduced significantly in Adavosertib, and Adavosertib + AZD6738 treated cells.

We also observed a slight increase in Cdk1 activity in AZD6738 and UCN-01 treated cells as compared to vehicle control. This might be due to the indirect role of ATR or Chk1 in regulating Cdk1 activity through Cdc25 (Figures 6D, E) (Qiu et al., 2018; Moiseeva et al., 2019). Yet in the absence of exogenous DNA damage causing agents, ATR/Chk1 signaling to the G2/M checkpoint is weak–as evidenced also by the small increase in cells entering mitosis after UCN-01 or AZD6738 treatment (Figure 5A). Consequently, Myt1 overexpression negligibly influences Cdk1 activity in AZD6738 or UCN-01 treated cells - particularly compared to Adavosertib or PD166285 treated cells (Figure 6F). Consistent with that observation, Myt1 overexpression did not influence significantly mitotic entry in cells treated for 4 h with AZD6738 or UCN-01. Based on the effect in these assays, indicating a less dominant role of Myt1 in regulating Cdk1 in the presence of Wee1, one could assume that Myt1 does not significantly affect cell sensitivity to ATR or Chk1 inhibitors. Yet we see exactly the opposite in two assays, the crystal violet assay (Figures 4B, C) measuring the number of live cells (i.e., a mixture of proliferation and survival assay) and the colony formation assay measuring clonogenic survival (Figures 4F, G). In both cases, but especially in the latter, Myt1 overexpression has a big effect on cancer cell sensitivity to either ATR (AZD6738) or Chk1 (UCN-01) inhibition. Unlike in the assays measuring Cdk1 activity after a short period of inhibitor treatment, in the survival/proliferation assays cells were treated with ATR or Chk1 inhibitor for days. The reduction of signaling in the ATR/Chk1 axis will significantly increase replication and genotoxic stress (i.e., DNA damage over time) leading to increased reliance of cellular survival on Wee1/Myt1. We previously showed the strong synthetic lethality between Wee1 and ATR inhibitors. The intricate cellular events leading to synergy between Wee1 and ATR inhibition over longer periods, such as during therapy, are discussed in detail in Bukhari et al. (2019); Bukhari et al. (2022) and we refer the reader to those manuscripts. Here we indicate that in the case of persistent inhibition of the ATR/Chk1 axis, Myt1 overexpression can increase cell survival. In other words, during prolonged inhibition of ATR or Chk1 (such as during clinical treatment) Myt1 levels can determine the drug sensitivity of cancer cells and, as a consequence, the efficacy in cancer therapy.

To test ATR activation–a readout of replication stress, we analysed lysates of cells treated with the above checkpoint kinase inhibitors for Chk1 S345 phosphorylation. As expected, AZD6738 abolished any detectable levels of phosphorylation at this ATR specific target site of Chk1. Of note and in agreement with previous literature (Domínguez-Kelly et al., 2011; Aarts et al., 2012; Duda et al., 2016; Moiseeva et al., 2019), Wee1 inhibition (by Adavosertib or PD0166285) led to strong ATR activation (Figure 5D). This could result from either Wee1 and ATR’s role in S phase (replication stress) or in mitosis (Aarts et al., 2012; Kabeche et al., 2018; Bukhari et al., 2019; Bukhari et al., 2022). Importantly, Myt1 overexpression strongly reduced ATR activation, indicating the protective role of Myt1 in the absence of Wee1 activity. As ATR is activated by single stranded DNA - structures arising from replication fork uncoupling, resection of double strand DNA breaks or R-loop formation (Gamper et al., 2013; Kabeche et al., 2018) - it is tempting to speculate that Myt1 overexpression represses replication stress caused by Wee1 inhibition and can partially compensate also for Wee1 loss in S phase. A role of Myt1 in modulating ATR activation in mitosis can also not be excluded. Further investigation on the interplay of Myt1/Wee1 and ATR/Chk1 in the various cell cycle phases are warranted to address these questions.

Since upregulation of Myt1 is a barrier towards cancer cell killing by G2/M checkpoint inhibitors, Myt1 inhibition is a potential step to subvert the resistance to Wee1 inhibition. Therefore, we investigated the cytotoxicity of PD166285, a Wee1/Myt1 dual inhibitor. Here, we show that treating HeLa and MDA-MB-231 cells with PD166825 causes decreased levels of the inhibitory phosphorylation at T14 and Y15 on Cdk1 (Supplementary Figure S2B), whereas Adavosertib only reduced the levels of Y15-Cdk1 (Supplementary Figure S2B). These data confirm previous studies that PD166285 inhibits both Wee1 and Myt1 whereas Adavosertib inhibits Wee1, but not Myt1 in cancer cells (Wang et al., 2001; Lewis et al., 2019). Given the dual specificity of PD166285 against both Wee1 and Myt1, we predicted that PD166285 would be able to induce higher Cdk1 activity in comparison to Adavosertib. It would also be a better agent to counteract the protective effect of Myt1 overexpression as compared to Adavosertib. However, at the same concentration PD166285 was found to be less effective than Adavosertib in activating Cdk1 as it increased Cdk1 activity by ∼3-fold as compared to 7-fold in Adavosertib treated cells (Figures 5B, C). Nevertheless, PD166285 has a similar IC50 than Adavosertib in HeLa cells (Figures 4A, D) and at the same concentration leads to an at least equal activation of ATR (Figure 5D). The discrepancies in drug efficacy in Cdk1 activation and cell killing by the two drugs could indicate that they have off targets influencing survival or that Myt1 inhibition contributes to cell killing not just via Cdk1 activation. Regarding the latter, Myt1 overexpression not only conferred resistance to Adavosertib, but also to PD166285 (Figure 6C).

In regard to the former, unintended off-target effects are a major limitation of several small molecule inhibitors. Adavosertib is reported to exhibit activity against several other kinases including Plk1, Yes, and Src (Hirai et al., 2009; Wright et al., 2017; Zhu et al., 2017). Similarly, PD166285 has been shown to exhibit activity against Chk1, Src, epidermal growth factor receptor (EGFR), fibroblast growth factor receptor 1 (FGFR1), and platelet-derived growth factor receptor b (PDGFRb) (Panek et al., 1997; Dimitroff et al., 1999; Wang et al., 2001; De Witt Hamer et al., 2011). The unintended inhibition of one or more of these kinases may influence the effects of Wee1 inhibition by Adavosertib or of Wee1/Myt1 inhibition by PD166285. Another observation of note is that PD166285 only partially inhibited Myt1 activity. At 250–500 nM, PD166285 only reduces pT14-Cdk1 levels by 40%–60% in HeLa and 50%–70% in MDA- MB-231 cells (Supplementary Figure S2B). Therefore, the pool of uninhibited Myt1 may be sufficient to protect some cells from premature mitosis in Wee1 inhibited cells. These results emphasize the need for a better dual inhibitor or a more selective Myt1 inhibitor which can be used in conjunction with other checkpoint kinase inhibitors. Of note, a highly selective inhibitor of Myt1 developed by Repare Therapeutics, RP-6306, which is currently in phase I/II trials presents an attractive agent for further investigation in combination with the DNA damage checkpoint kinase inhibitors (Gallo et al., 2022).

Complicating the discussion are reports indicating that Myt1 kinase activity might not be required for Cdk1 inhibition in cells that overexpress Myt1. Wells et al. reported that overexpression of either wild-type or catalytically inactive Myt1 (Myt1 D251A) equally arrested HeLa cell populations in G2 phase (Wells et al., 1999). This G2 arrest was attributed to the ability of Myt1 to sequester Cdk1/cyclin B at the Golgi and endoplasmic reticulum independent of Cdk1 phosphorylation (Wells et al., 1999). Cytoplasmic sequestration of a non-phosphorylatable Cdk1 mutant (Cdk1 T14A/Y15F) was also reported to maintain G2 arrest in U-2 OS cells (Heald et al., 1993). If the kinase activity were not required for Myt1 to inhibit Cdk1, then small molecule Myt1 kinase inhibitors are unlikely to exhibit a strong effect on cell cycle progression. Yet more recently a report indicated that kinase activity is essential for Myt1 to prevent premature mitosis as demonstrated with RP-6306, the Myt1 selective inhibitor recently developed (Gallo et al., 2022). In future experiments, it will be important to validate whether Myt1 kinase activity is specifically required to inhibit Cdk1 activity or just has a dominant effect over direct sequestration of Cdk1/cyclin B1. This could be tested by using RP-6306 and Adavosertib in monotherapy and combination and then analyzing Cdk1 activity in all the scenarios.

In conclusion, emerging evidence including our current findings, suggest that Myt1 is an important therapeutic target (Toledo et al., 2015; Lewis et al., 2019; Long et al., 2020; Shao et al., 2021; Gallo et al., 2022; Li et al., 2023) to avoid resistance or to synergize with drugs targeting checkpoint kinases entering the clinic. Therefore, it is a critical unmet need to evaluate selective Myt1 inhibitors both in stand-alone as well as combination therapy settings.

## Data Availability

The original contributions presented in the study are included in the article/Supplementary Material, further inquiries can be directed to the corresponding authors.

## Footnotes

1
https://clinicaltrials.gov/

## Supplementary material

SUPPLEMENTARY FIGURE S1 Tetracycline induced GFP-Myt1 expression in HeLa Flp-In T- REx cells. **(A)** Non treated and tetracycline-treated HeLa cells were fixed and stained for DAPI and GFP-Myt1. **(B)** HeLa cells were also analyzed by immunoblot for total levels of Myt1, GFP, and tubulin. Long exposure shows endogenous Myt1 levels. Myt1 overexpression does not induce a cell cycle arrest: **(C)** HeLa cells were either not treated or treated with 2 μM tetracycline for 48 h and then analyzed for total levels of PH3 by immunofluorescence microscopy. Scale bar = 20 μm. **(D)** Donut charts show the portion of PH3 positive to DAPI-stained nuclei. The number of cells counted for each treatment is presented in the centre of each plot. **(E)** Representative interphase and mitotic cells (at indicated stages) expressing GFP-Myt1 are shown. Scale bar = 20 μm. Experiments were repeated three times.

SUPPLEMENTARY FIGURE S2 Myt1 overexpression prevents premature mitosis in the presence of Adavosertib and Adavosertib+AZD-6738 treatment. **(A)** HeLa cells were seeded on coverslips in the presence or absence of tetracycline (2 μM) for 24 h. Cells were synchronized in G1-S phase by a single thymidine block. Following synchronization cells were released into media containing kinase inhibitors or DMSO for 4 h. Cells were stained for DNA and PH3 and analyzed by immunofluorescence microscopy. Scale bar = 10 μm. Experiment was repeated three times. PD-166285 inhibits Cdk1 phosphorylation on T14 and Y15: **(B)** HeLa and MDA MB-231 cells were treated with indicated concentrations of Adavosertib (Adav) or PD0166285 (PD) for 4 h and then analyzed for total levels of pY15-Cdk1, pT14-Cdk1, and Cdk1 by immunoblot. The quantitation of pT14- and pY15-Cdk1 relative to total Cdk1 levels is presented below the respective immunoblots. For HeLa, quantitation reflects an average of 2 experiments whereas for MDA-MB-231 the quantitation is from a single experiment.
