# Supplementary figures and images for: Myt1 overexpression mediates resistance to cell cycle and DNA damage checkpoint kinase inhibitors
Source: Front Cell Dev Biol. 2023 Nov 2;11:1270542. doi: 10.3389/fcell.2023.1270542 (PMC10652759; doi:10.3389/fcell.2023.1270542)

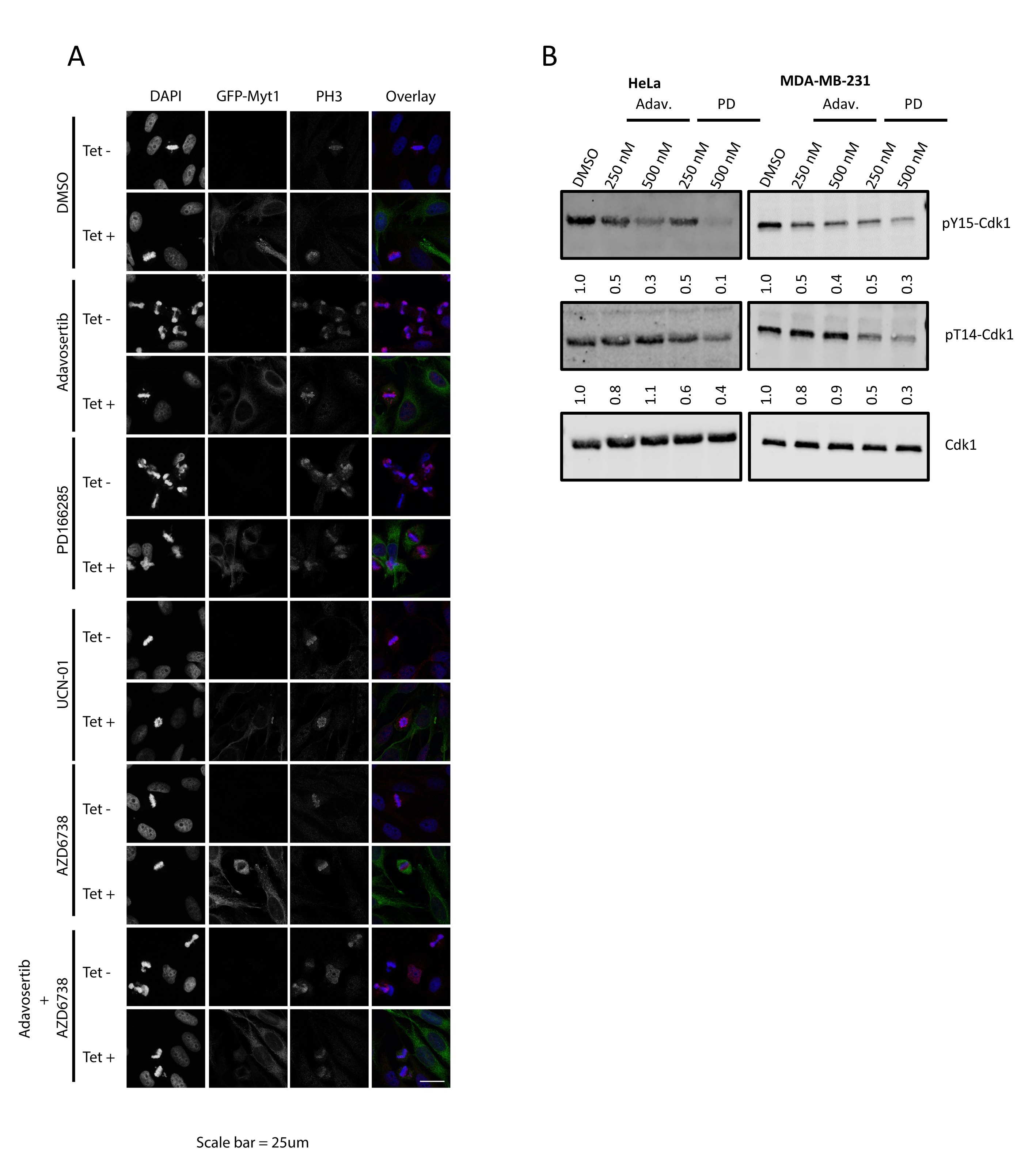

Supplement: Supplementary file 1 [file Image2.TIF]

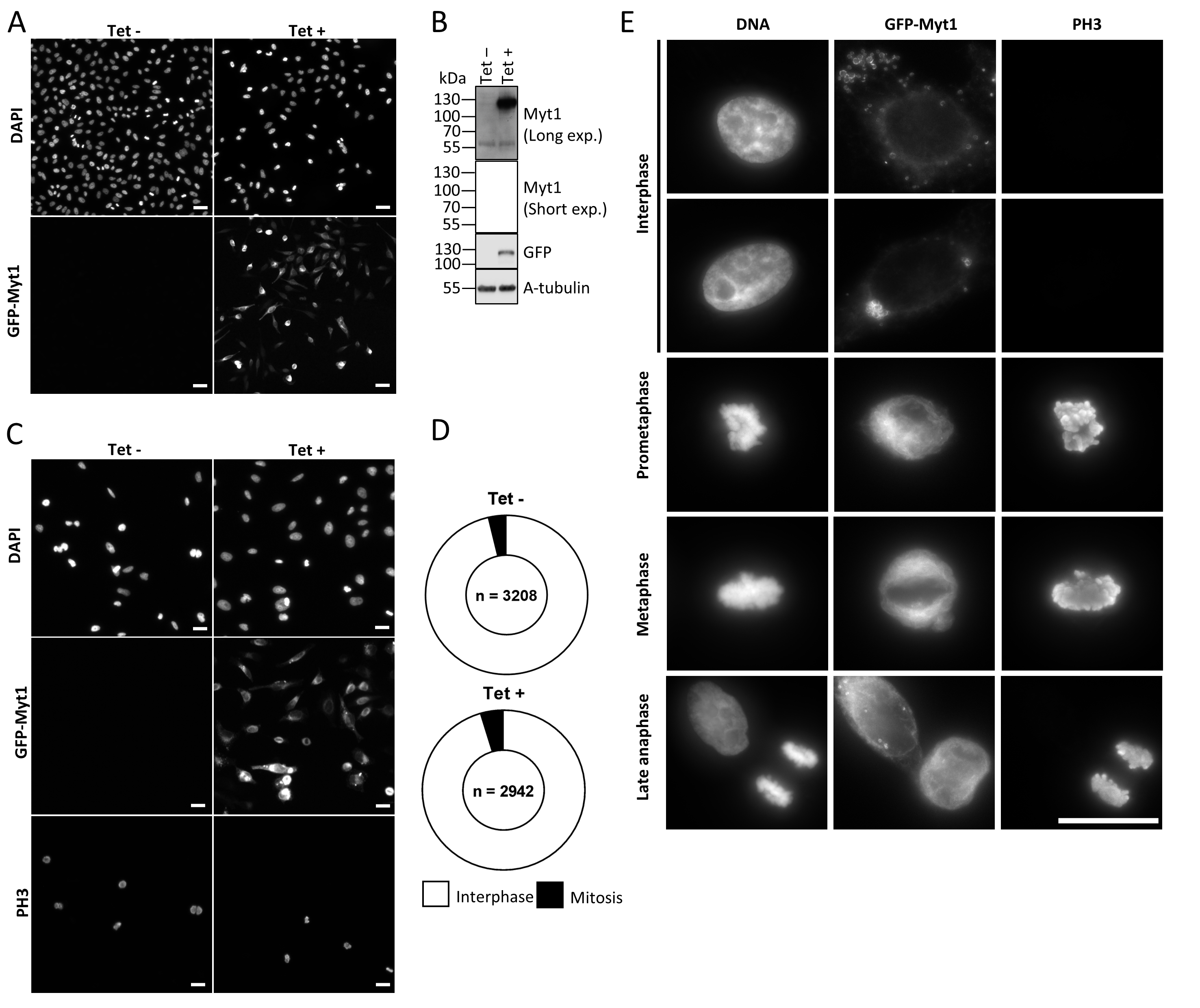

Supplement: Supplementary file 2 [file Image1.TIF]
